# Nuclear BMI-1 expression in laryngeal carcinoma correlates with lymph node pathological status

**DOI:** 10.1186/1477-7819-10-206

**Published:** 2012-10-02

**Authors:** Eugenia Allegra, Lidia Puzzo, Valeria Zuccalà, Serena Trapasso, Enrico Vasquez, Aldo Garozzo, Rosario Caltabiano

**Affiliations:** 1 Department of Otolaryngology-Head and Neck Surgery, University of Catanzaro, Catanzaro, Italy; 2 Department of Pathology, University of Catania, Catania, Italy; 3 Department of Pathology, University of Catanzaro, Catanzaro, Italy

**Keywords:** BMI-1, Laryngeal cancer, Lymph node metastasis, Squamous cell carcinoma

## Abstract

**Background:**

The main cause of treatment failure and death in laryngeal squamous cell carcinoma is metastasis to the regional lymph nodes. The current clinical staging criteria fail to differentiate patients with occult metastasis from patients without metastasis. Identifying molecular markers of the disease might improve our understanding of the molecular mechanisms underlying the pathogenesis and development of laryngeal carcinoma and may help improve clinical staging and treatment.

**Methods:**

Sixty-four previously untreated patients who underwent surgical excision of laryngeal squamous cell carcinoma with neck dissection were included in this study. The expression of B cell-specific Moloney murine leukemia virus integration site 1 (BMI-1) was examined immunohistochemically on formalin-fixed paraffin-embedded primary tissue specimens.

**Results:**

Nuclear expression of BMI-1 (nBMI-1) was detected in 32 of the 64 tumors (50%), cytoplasmic expression of BMI-1 (cBMI-1) was detected in 22 (34.4%), and 10 tumors (15.6%) showed no BMI-1 immunoreactivity. High nBMI-1 expression levels (≥10) were detected in 28 of the 32 (87.5%) nBMI-1-positive patients. Multivariate analysis including age at diagnosis, grade, tumor location, TNM status, and nBMI-1 expression showed that a high nBMI-1 expression level was an independent prognostic factor for lymph node metastasis.

**Conclusion:**

The expression of BMI-1 in patients with laryngeal carcinoma seems to correlate with lymph node metastasis.

## Background

Laryngeal squamous cell carcinoma is the most common type of head and neck cancer, and its incidence rate in Italy is approximately 5,000 new cases per year [1]. Most patients affected by laryngeal cancer are in the locoregionally advanced stage of disease at the time of diagnosis. The main cause of treatment failure and death is metastasis to the regional lymph nodes and distant metastasis [2]. The current clinical staging criteria fail to differentiate patients with occult metastasis from patients without metastasis. Many investigators are currently working to identify molecular markers of the disease to improve our understanding of the molecular mechanisms underlying the pathogenesis and development of laryngeal carcinoma and to improve its clinical staging.

Recently, B cell-specific Moloney murine leukemia virus integration site 1 (BMI-1) seems to be a new putative marker for head and neck solid tumors.

BMI-1 is an essential constituent of the polycomb repressive complex-1, a key epigenetic regulator. BMI-1 controls the cell cycle and self-renewal of tissue stem cells by regulating chromatin and histone structures. BMI-1 was identified initially as an oncogene that cooperates with c-Myc in the generation of mouse pre-B cell lymphomas, and it has been implicated in the maintenance of self-renewal of neural, hematopoietic, and intestinal stem cells [3]. BMI-1 protein, together with c-Myc protein, regulates the INK4a/ARF locus, which encodes two unrelated tumor suppressors, p16^INK4a^ and p19ARF, which act in the two main cell cycle control pathways (pRb and p53, respectively). BMI-1 is highly expressed in breast cancer [4], colorectal cancer [5], prostate cancer [6], metastatic melanoma [7] and non-small cell lung cancer [8]. At this time very few studies have been made on BMI-1 expression in head and neck tumors. Because of discordant results with regard to nasopharyngeal carcinoma [9], oral squamous cell carcinoma [10] and laryngeal carcinoma [11] no definitive conclusions can be made.

The objective of this study was to evaluate immunohistochemically the expression of BMI-1 in laryngeal carcinomas and to determine its clinical significance.

## Methods

### Patients

Previously untreated patients who underwent surgical excision of laryngeal squamous cell carcinoma with neck dissection in the Department of Otolaryngology-Head and Neck Surgery ENT ‘Magna Graecia’ University of Catanzaro between January 2004 and December 2009 were considered for this study. From the original 72 patients, eight were subsequently excluded because of the poor quality of the histopathological sample available for review or loss to follow-up. Sixty-four patients were included in this study.

Fifty-eight of the 64 patients were men and six were women; their median age was 67 ± 13.0 years (range 43 to 86 years). Twenty-three (35.9%) of the 64 patients had a glottic localization and 41 (64.1%) had a supraglottic localization. The histological grade was G1 in 8 cases, G2 in 36, and G3 in 18. All were stage III–IV. According to the TNM classification, the stages of the primary tumors were T2 in 24 patients, T3 in 31, and T4 in 9. Forty-seven patients underwent a total laryngectomy, 12 had a supraglottic laryngectomy, and 5 had a supracricoid laryngectomy. The clinical classification of the regional lymph nodes was N0 in 11, N1 in 22, N2a in 13, N2b in 14, and N2c in 4 patients. Twenty-eight patients had unilateral neck dissection and 36 had bilateral neck dissection. A total of 100 hemineck dissections were performed; 42 were selective (Levels II to IV) and 58 were modified (type II to III). Pathological examination showed that 33 of the 64 patients (51.5%) had lymph node metastasis. All of these patients underwent postoperative radiation.

All patients included in this study were followed up every three months; the median clinical follow-up was 36 ± 21.5 months.

The protocol was approved by the Institutional Review Board of ‘Magna Graecia’ University of Catanzaro. All patients were informed before giving informed consent.

### Immunohistochemistry

The specimens derived from primary tumors were fixed in 10% buffered formalin, processed routinely, and embedded in paraffin. Four-micrometer-thick sections were stained with H & E for histological evaluation of the specimens. Additional sections were prepared for immunohistochemical analysis. The immunohistochemical assay was performed on 4 μm-thick sections cut from the blocks and float-mounted on Super Frost/Plus slides. Slides were deparaffinized and rehydrated through xylene and graded ethanol to water. Antigen retrieval was performed using a heat-induced method in which the specimens were placed in a citric acid solution (Target Retrieval Solution, pH 6.1; Dako Cytomation, Glostrup, Denmark) for 30 min at 94°C in a vegetable steamer and then brought to room temperature within 20 min. All slides were quenched for 5 min in 0.03% hydrogen peroxide (EnVision + System-HRP (DAB); Dako Cytomation) to block endogenous peroxidase. The primary antibody, mouse anti-human BMI-1 antibody (R&D Systems, Minneapolis, MN, USA) diluted 1:100), was applied for 60 min in a moist chamber. After washing in Tris-buffered saline (TBS) at pH 7.6, the MACH 4 Universal HRP-Polymer kit (Biocare Medical, Concord, CA, USA) was applied for 60 min as the detection system. The sections were washed again in TBS. Staining was completed with a 10 min incubation with 3,3′-diaminobenzidine substrate chromogen (Dako Cytomation). Slides were counterstained in hematoxylin, dehydrated through graded ethanol solutions, and mounted using an aqueous medium (Faramount, Dako Cytomation).

The staining intensity of BMI-1 was evaluated as negative (0), weak (1), moderate (2), or strong (3), as described previously [11]. The percentage of positive cells was used to classify scores for specimens as follows: negative (score 0); 1% to 10% stained cells (score 1); 11% to 30% stained cells (score 2); 31% to 50% stained cells (score 3); 51% to 80% stained cells (score 4); and >80% stained cells (score 5). The staining intensity was multiplied by the percentage of positive cells to obtain an intensity reactivity score (IRS). Patients were classified into three groups based on the IRS: IRS = 0 (no expression), IRS <10 (low expression), and IRS ≥10 (high expression) [12]. Nuclear or cytoplasmic BMI-1 expression was also evaluated.

### Statistical analysis

Statistical analysis was performed with MedCalc software (Mariakerke, Belgium) using the chi-squared test and Fisher’s exact test. All reported *P* values are two-tailed and *P <*0.05 was considered significant. Overall survival (OS) and disease-specific survival (DSS) were calculated according to the Kaplan–Meier method. Multivariate analysis was performed to identify independent prognostic factors using multiple regression analysis.

## Results

**Figure 1:**
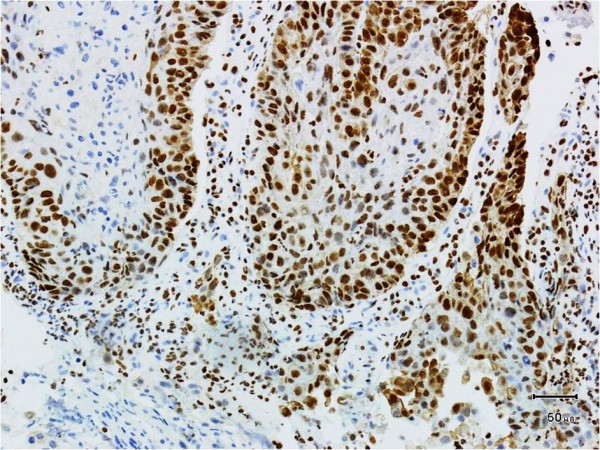
**The expression of BMI-1 protein in laryngeal carcinoma.** High nuclear BMI-1 expression (SP 200×).

Nuclear expression of BMI (nBMI-1) in tumor cells was detected in 32 of the 64 tumors (50%) (Figure 1), cytoplasmic expression of BMI-1 (cBMI-1) was detected in 22 (34.3%), and 10 tumors (15.6%) showed no BMI-1 immunoreactivity. High levels (IRS ≥10) of nBMI-1 expression were detected in 28 of the 32 (87.5%) nBMI-1-positive patients. The patients’ clinical data were stratified according to nBMI-1 expression into a negative or low (0 to 10) group and a high nuclear expression (≥10) group (Table 1). High expression of nBMI-1 was detected in 39.1% of the glottic tumors and in 46.2% of the supraglottic tumors, although this difference was not significant (*P* = 0.61). There was also no significant correlation between nBMI-1 expression and histological grade (*P* = 0.38). High expression of nBMI-1 was detected in 33.3% of the T2 tumors, in 38.7% of the T3 tumors, and in 88.8% of the T4 tumors (*P* = 0.01). Four of the 11 (36.3%) patients with clinically negative nodes showed high nBMI-1 expression and 29/53 (54.7%) of those with clinically positive nodes showed high expression of nBMI-1 (*P* = 0.74). None of the 31 patients with negative pathological nodes showed high nBMI-1 expression in their primary tumors. By contrast, 84.8% (28/33) of those with positive pathological nodes showed high expression of nBMI-1 (*P <*0.0001).

**Table 1:** Correlation between nBMI-1 expression and clinicopathological data

**Clinical data**	**N**	**BMI-1 nuclear expression negative or**	
		**low**	**high**
Localization			
Glottic	23	14	9
Supraglottic	41	22	19
			*P* = 0.61
Histological grade			
G1	8	6	2
G2	36	20	16
G3	18	10	8
			*P = 0.38
T classification			
T2	24	16	8
T3	31	19	12
T4	9	1	8
			*P = 0.01
N clinical status			
N0	11	7	4
N+	53	29	24
			*P*= 0.74
N pathological status			
N0	31	31	0
N+	33	5	28
			*P* = 0.0001

*Chi-square test. nBMI-1, nuclear BMI-1.

The multivariate analysis included age at diagnosis, grade, tumor location, T and N classification, TNM status, and nBMI-1 expression. A high expression of nBMI-1 was an independent prognostic factor for lymph node metastasis (*P* = 0.0002).

**Figure 2:**
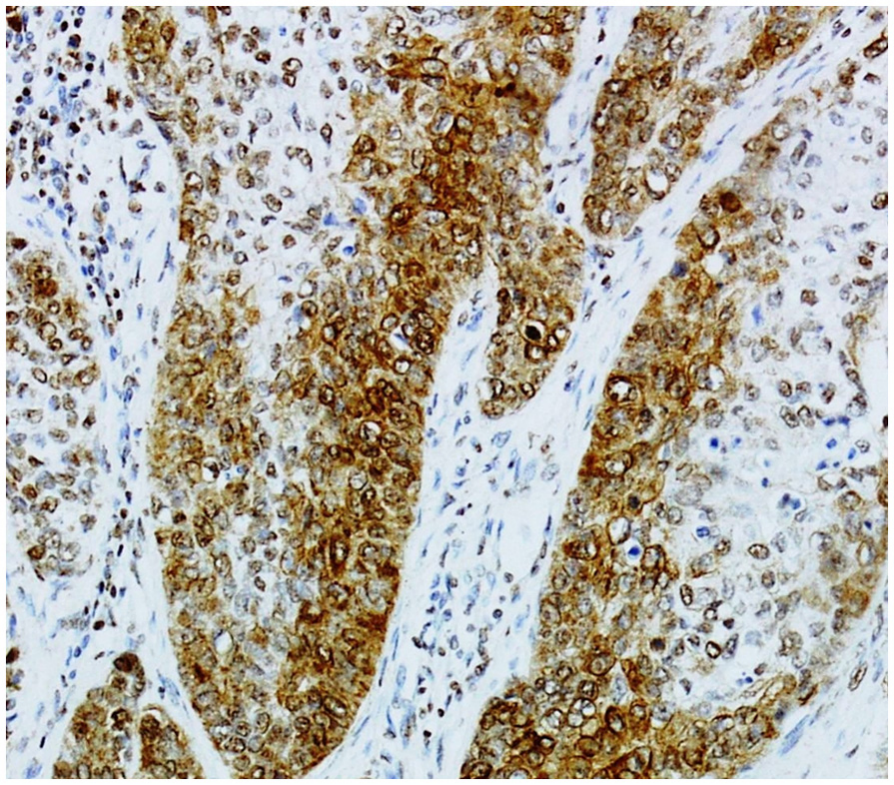
**The expression of BMI-1 protein in laryngeal carcinoma.** High cytoplasmatic BMI-1 expression (SP 200×).

Cytoplasmic staining for BMI-1 was detected in 22 of the 64 primary tumors; 9 of these (40.9%) showed a high expression level (≥10) (Figure 2). None of the 33 primary tumors associated with metastatic lymph nodes showed cytoplasmic immunoreactivity for BMI-1. However, six of the nine patients with high levels of cBMI-1 died of distant metastasis.

High cBMI-1 expression correlated significantly with distant metastasis (*P* <0.05), and negative or low cBMI-1 expression correlated with negative lymph nodes (*P <*0.05) (data not shown).

**Figure 3:**
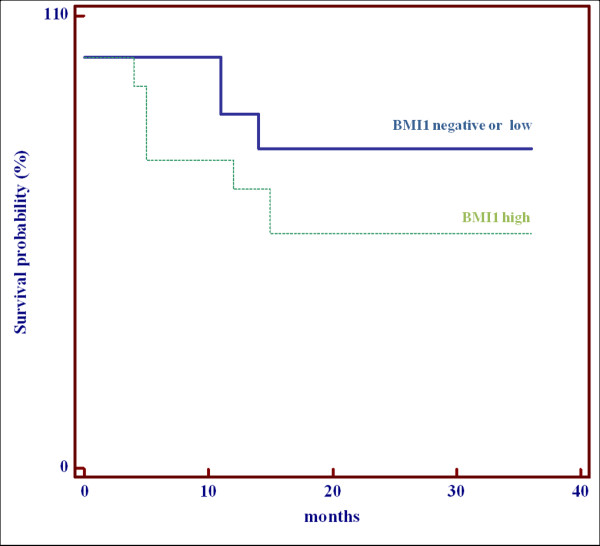
**Disease-specific survival.** The three-year specific survival in the cohort of patients expressing no or a low level of nBMI-1 compared with patients with high expression of nBMI-1 (*P* = 0.058). nBMI-1, nuclear BMI-1.

The three-year OS and DSS of the 64 patients were 57.8% and 68.7%, respectively. The three-year specific survival was 77.7% (28/36) in the cohort of patients expressing no or low three-year specific survival was 77.7% (28/36) in the cohort of patients expressing no or low levels of nBMI-1 (0 to 10) and 57.1% (16/28) in the group of patients with high nBMI-1 expression (*P* = 0.058) (Figure 3). Multivariate analysis showed no significant correlation between high nBM-I expression and poor survival (*P* = 0.48, significance level *P* = 0.24).

In the patients with no or low expression of nBMI-1, the specific cause of death was distant metastasis in all cases. In the patients with high expression of nBMI-1, the specific cause of death was progression of the primary tumor in two patients, recurrence of lymph node metastasis in six patients, and distant metastasis in four patients.

## Discussion

Lymph node metastasis represents the most adverse clinical prognostic factor and decreases OS by about 50% [13].

Clinical determination of lymph node metastasis made by palpation, computed tomography (CT), and magnetic resonance imaging (MRI) has a sensitivity of 38% to 78% for occult metastatic lymph nodes [14,15] and a 7% to 37% probability of metastatic lymph nodes in patients classified with N0 disease [16]. To improve the choice of the modality of treatment, immunohistochemical and molecular prognostic markers should be useful for identifying patients with occult metastasis at diagnosis [17].

In this study, we report a correlation between the clinical data at diagnosis and BMI-1 expression in primary tumors from patients affected by laryngeal carcinoma. The most relevant finding from the study was the significantly high nBMI-1 expression detected in primary tumors from patients with metastatic lymph nodes.

A previous study by Chen *et al*. [11] on 20 laryngeal carcinomas reported a higher expression of BMI-1 correlated with advanced stage of disease and well differentiated histological grade. A correlation between T and N classification and prognostic significance was not reported in this study. However, the higher expression of BMI-1 in the advanced stage of disease can be due, in agreement with our previous results [18], to the T and N status of the patients classified as advanced clinical stage.

In our study the expression of nBMI-1 correlated significantly with T classification and pathological N status. The multivariate analysis showed that nBMI-1 expression was the sole independent prognostic factor for lymph node metastasis, although it failed as an independent prognostic factor of poor DSS. This suggests that the expression of BMI-1 is a reliable marker of the presence of lymph node metastases at diagnosis. We detected cBMI-1 expression in 34.3% of the primary laryngeal tumors. cBMI-1 expression did not correlate with lymph node metastasis, but high cBMI-1 expression correlated significantly with distant metastasis (*P* = 0.001).

The clinical implications of BMI-1 expression in head and neck tumors are unclear because there are few clinical studies. Hayry *et al*. [19] reported that negative nBMI-1 expression seems to correlate with poor recurrence-free survival at two years in early tongue squamous cell carcinomas (T1–T2N0). They found nBMI-1 positivity in 82% of cases, but they did not report any cytoplasmic expression. They also reported that nBMI-1 expression was a prognostic marker in patients undergoing elective neck dissection.

Huber *et al*. [12] studied tumors of the oropharynx and oral cavity and reported that 21.4% of tumors were positive for nBMI-1 and 27% for cBMI-1. In their study, cytoplasmic positivity was inversely related to OS and DSS, but this correlation was significant only for cancers of the oropharynx. The DSS was significantly better in patients with oropharyngeal cancer with low levels of nBMI-1, although the results of the multivariate analysis were not significant.

Our data for laryngeal squamous cell carcinoma agree with the findings of Huber *et al*. and Hayry *et al*. [19] showing that nBMI-1 does not correlate with OS or with specific survival. Furthermore, some common elements emerge. These earlier studies and our study suggest that nBMI-1 is related to the capacity for lymph node metastasis, whereas cBMI-1 appears to correlate with the capacity for distant metastases.

In the study by Hayry *et al*. [19], tongue tumors were all early stage and expressed only nuclear staining, which correlated with the N state but not with the prognosis. By contrast, Huber *et al*. [12] found greater cytoplasmic expression than nuclear expression, and a correlation between cytoplasmic expression and poor DSS. This suggests that the nuclear and cytoplasmic expression of BMI-1 have different roles in the progression of tumors of the head and neck. This may explain why nuclear and cytoplasmic BMI-1 expression levels were not significant independent predictors of OS or DSS when considered separately in the multivariate analysis. Nuclear expression appears to play a significant role in the lymph node-metastasizing capacity, whereas cytoplasmic expression appears to play a role in the ability to metastasize to distant sites.

Recent evidence shows that head and neck tumor growth and progression depend on a subset of cells defined as ‘cancer stem cells’ [20,21] that possess the ability for self-renewal within a tumor expressing different phenotypes, which are responsible for resistance to chemo-radiotherapy and the capacity to metastasize locally or to distant sites.

In laryngeal carcinomas in particular, it has been revealed that cancer stem cells expressing the isoform v3 of CD44 surface antigen seem to have the ability for lymph node metastasis while those expressing the isoform v6 of CD44 seem to have ability for distant metastasis [22].

BMI-1 is considered to be a stem cell-related gene that is implicated in the tumorigenesis of head and neck tumors [21]; its nuclear or cytoplasmatic expression could be related to the cancer stem cell phenotype in which it is expressed. However, it is not yet known whether the nuclear and cytoplasmic expression of BMI-1 varies between different subsets of stem cells. The correlation found in this study between nBMI-1 expression and pN staging suggests that BMI-1 is a potential candidate marker in treatment planning for the neck in a subset of patients with clinically N0 laryngeal cancer. Further studies are needed to confirm our hypothesis.

## Conclusions

The expression of BMI-1 in patients with laryngeal carcinoma seems to be a potential marker of tumor aggressiveness. A high BMI-1 expression may detect lymph node metastasis at diagnosis and can be useful in a subset of patients to decide on neck treatment.

However, further and larger studies are needed to confirm our results.

## Abbreviations

BMI-1: B-cell specific Moloney murine leukemia virus integration site 1; cBMI-1: cytoplasmic expression of BMI-1; DSS: disease-specific survival; H & E: haematoxylin and eosin; IRS: intensity reactivity score; nBMI-1: nuclear expression of BMI-1; OS: overall survival; TBS: Tris-buffered saline.

## Competing interests

The authors declare that they have no competing interests.

## Authors’ contributions

EA contributed to the study concept, study design and writing of the manuscript. LP contributed to the study design and performed the experiments. VZ performed the experiments. ST obtained the patients’ data and conducted the statistical analysis. EV contributed to data interpretation. AG reviewed the manuscript. RC performed experiments and data analysis. All authors read and approved the final manuscript.
